# Epigenetic Inactivation of RIPK3-Dependent Necroptosis Augments Cisplatin Chemoresistance in Human Osteosarcoma

**DOI:** 10.3390/ijms26083863

**Published:** 2025-04-18

**Authors:** Aditya Sharma, Daniel Pettee, Christine Mella, Catherine Hord, Maximilian Brockwell, Samantha Hardy, Hope C. Ball, Fayez F. Safadi, Steven J. Kuerbitz

**Affiliations:** 1 Division of Hematology Oncology, Akron Children’s Hospital, One Perkins Square, Akron, OH 44308, USA; adityasharma@llu.edu (A.S.); dfpettee@gmail.com (D.P.); cmella@akronchildrens.org (C.M.); sjkuerbitz@gmail.com (S.J.K.); 2 College of Medicine, Northeast Ohio Medical University, 4029 State Route 44, Rootstown, OH 44272, USA; chord1@neomed.edu (C.H.); mbrockwell@neomed.edu (M.B.); shardy@neomed.edu (S.H.); fsafadi@neomed.edu (F.F.S.); 3 Rebecca D. Considine Research Institute, Akron Children’s Hospital, One Perkins Square, Akron, OH 44308, USA; 4 University Hospitals Health Systems, 11100 Euclid Ave, Cleveland, OH 44106, USA

**Keywords:** osteosarcoma, RIPK3, CpG islands, chemoresistance, necroptosis

## Abstract

Osteosarcoma (OS) is the most common primary bone malignancy in children and adolescents. Unfortunately, drug resistance limits the efficacy of chemotherapeutic treatment and compromises therapeutic outcomes in a substantial proportion of cases. Aberrant CpG island methylation-associated transcriptional silencing contributes to chemoresistance in pediatric solid tumors. Here, using whole-genome DNA methylation screening on 16 human primary OS specimens, we identify receptor interacting protein kinase-3 (RIPK3), a molecular regulator of the necroptosis programmed cell death pathway, as a gene target of aberrant CpG methylation and demonstrate its role in human OS chemoresistance. We validated these findings via enforced expression and DsiRNA silencing, and evaluated the role of RIPK3 in cisplatin chemosensitivity and necroptosis activation through MLKL phosphorylation. We found that CpG island methylation results in RIPK3 silencing in primary human OS samples and cell lines. Enforced RIPK3 expression significantly enhanced cisplatin cytotoxicity in OS cells and DsiRNA knockdown reversed the cisplatin-sensitive phenotype. In cells with enforced RIPK3 expression, cisplatin treatment significantly increased phosphorylation of both RIPK3 and its target, MLKL, indicative of induction of necroptosis. Here, we identify RIPK3 as an important mediator of chemoresistance in OS and a potential pharmacologic target to improve chemotherapy efficacy in drug-resistant tumors.

## 1. Introduction

Osteosarcoma (OS) is a high-grade malignancy of bone-forming cells, likely derived from mesenchymal progenitor cells. While comparatively rare, OS is the most common primary bone malignancy in children and is especially prevalent in adolescents [1,2]. OS occurs most frequently at the metaphysis of the long bones, particularly those of the lower limb adjacent to the knee [1,3]. To date, a consensus understanding of the molecular mechanisms by which mesenchymal progenitor cells transform into osteoid producing cancer cells has not emerged. Evidence implicates multiple genetic targets and pathways comprising a complex signaling network in the bone microenvironment [4]. Recent genetic studies have uncovered OS-associated germline mutations in genes regulating cell cycle and DNA damage repair mechanisms. These include tumor protein 53 (*TP53*), retinoblastoma protein 1 (*RB1*), *MYC* proto-oncogene, and cyclin-dependent kinase inhibitor 2A (*CDKN2A*) [5,6,7,8]. However, the frequency of these mutations is relatively rare (18–28%) and the majority of OS cases arise sporadically [9,10]. The lack of common genetic risk factors and the degree of genetic heterogeneity between OS patients complicates the development of novel therapeutics for the treatment of high-grade OS.

Mainstay treatment strategies for high-grade OS typically include surgical excision of the primary tumor and neoadjuvant/adjuvant chemotherapy employing the agents methotrexate, adriamycin (doxorubicin), and platinum (cisplatin; together referred to as MAP) [11,12]. The efficacy of both of these modalities is critical to successful treatment [4]. Tumor resistance to MAP therapy, however, compromises therapeutic efficacy in a substantial proportion of cases, resulting in poor clinical outcomes [13,14,15]. To date, effective alternative salvage regimens for relapsed and chemo resistant OS have not been identified [4].

Dysregulation of gene expression by aberrant DNA methylation and other epigenetic processes is well established as a mechanism of tumor progression [16,17,18]. Aberrant DNA methylation, including CpG island methylation-associated silencing of target genes, has also been shown to affect chemoresistance in pediatric solid tumors including medulloblastoma, neuroblastoma, and OS [19,20]. We therefore sought to identify gene targets of aberrant CpG island methylation-associated transcriptional silencing relevant to the chemoresistance phenotype in human OS. To this end, we first performed genome-wide methylation profiling on a panel of human OS primary tumors compared to a panel of control specimens. In analyzing the data for gene-associated CpG islands exhibiting higher than expected methylation, given the overall prevalence of CpG methylation genome-wide in the tumor sample set, we identified the 5′ CpG island of necroptosis-associated receptor interacting protein kinase-3 (*RIPK3*) gene in the group demonstrating the highest level of aberrant methylation.

Necroptosis is a form of programmed cell death that is known to contribute to the pathology of inflammatory and neurodegenerative disorders such as idiopathic pulmonary fibrosis (IPF), multiple sclerosis (MS), amyotrophic lateral sclerosis (ALS), and Alzheimer’s disease (AD) [21,22,23,24,25]. Necroptosis is associated with the onset, progression, and chemo resistance of various cancers such as acute myeloid leukemia (AML), breast, and colorectal cancer [26,27,28]. Unlike apoptosis, necroptosis results in the release of pro-inflammatory damage-associated molecular patterns (DAMPs) and the induction of tumor necrosis factor alpha (*TNF-α*), both associated with increased invasion and metastatic capacity [29,30,31,32]. RIPK3 is a RIP homotypic interaction motif (RHIM)-containing protein known to initiate necroptotic programmed cell death through a variety of signaling pathways such as death receptor, Toll-like receptor, and interferon gamma receptors following inflammation or pathogenic invasion [33]. Pathway activation leads to recruitment of other RHIM-containing proteins and activation of mixed-lineage kinase domain-like (MLKL) protein via serine phosphorylation [34]. Once activated, MLKL undergoes a transformational change that permits MLKL translocation from the cytosol to cellular and plasma membranes, where it results in loss of membrane integrity and cell death [35].

RIPK3 has been shown to induce necroptosis and the loss of RIPK3 expression has been shown to desensitize tumors to cisplatin cytotoxicity [36,37,38]. Here, we sought to investigate a potential role for *RIPK3* transcriptional silencing in cisplatin resistance in human OS. Development and implementation of pharmacologic strategies to reverse methylation-associated silencing of *RIPK3* may benefit a substantial proportion of human OS patients exhibiting the chemo resistant phenotype, even in the absence of novel cytotoxic drugs.

## 2. Results

### 2.1. The RIPK3 CpG Island Exhibits Aberrant Methylation in OS Cell Lines and Tumor Samples

**Figure 1:**
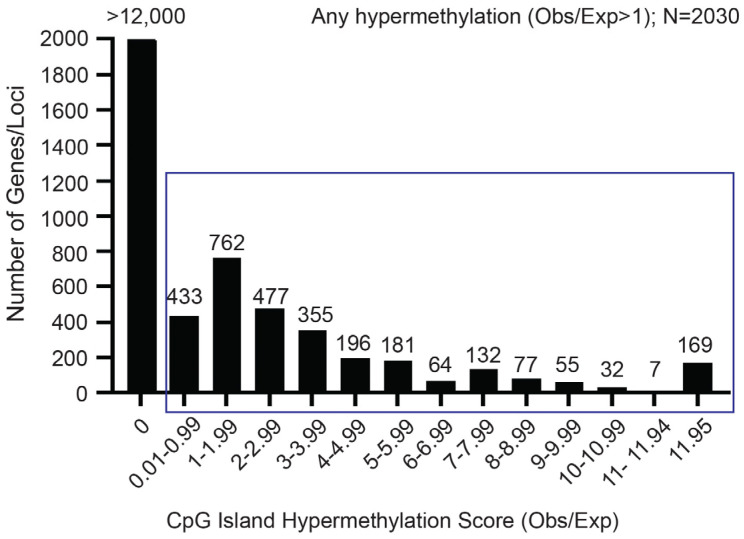
Hypermethylation of CpG islands is prevalent in human primary osteosarcoma (OS) tumor samples compared to levels detected in normal bone and primary human osteoblasts.

To identify potential mediators of chemoresistance in human OS, we performed genome-wide CpG methylation analysis across 16 human primary OS specimens compared to non-neoplastic control samples including human mesenchymal stem cell lines, normal adult skeletal muscle, bone, fetal liver, fetal brain, and adult adipose. Employing an analysis to identify CpG island targets exhibiting higher-than-expected prevalence of CpG methylation, given the overall level of CpG methylation, we identified approximately 3000 (of >15,000) CpG islands exhibiting such higher-than-expected methylation prevalence (Figure 1). Among the 169 most highly methylated CpG islands, we identified the *RIPK3* 5′ CpG island. As RIPK3 is the molecular regulator of the necroptosis programmed cell death pathway and has previously been observed to be downregulated in human OS samples, we examined the methylation patterns of *RIPK3* via DNA pyrosequencing and next-generation sequencing (Table 1) [39,40]. High level aberrant *RIPK3* CpG island methylation was observed in all (5/5) human OS cell lines. Similarly, markedly elevated levels of *RIPK3* methylation were identified in 6 of 11 primary OS tumor samples compared to normal bone and human osteoblasts (Table 1 and Table S1).

### 2.2. Aberrant CpG Island Methylation Is Associated with Downregulation of RIPK3 and MLKL Expression in Human OS Cell Lines

**Figure 2:**
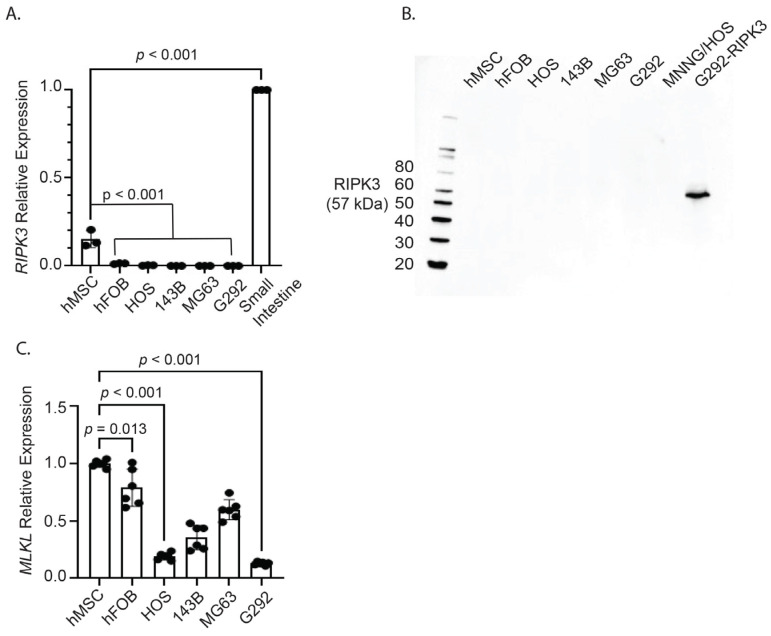
Transcription and translation of RIPK3 is inhibited in OS cell lines. (**A**) qPCR demonstrates *RIPK3* mRNA levels are significantly reduced compared to small intestine positive control. (**B**) Western blot analysis demonstrates RIPK3 expression is absent in OS cell lines HOS, 143B, MG63, G292, and MNNG/HOS, in hMSCs, and hFOBs. G292 RIPK3 transfected cells were used as a positive control. (**C**) Levels of MLKL, measured via qPCR, were also significantly reduced in OS cell lines compared to hMSCs and hFOBs. RIPK3; 57 kDa.

After identifying aberrant methylation patterns of *RIPK3* in OS primary tumor samples, quantitative real time PCR (qRT-PCR) analysis (*HPRT* was the normalizing control) found minimal to absent *RIPK3* mRNA expression in the OS cell lines compared to the small intestine tissue control (Figure 2A). Next, Western blot analysis was utilized to assess RIPK3 protein levels in several established OS cell lines (HOS, 143B, MG63, G292, and MNNGHOS), human mesenchymal stem cells (hMSCs), and human fetal osteoblasts (hFOB) [41,42]. Human small intestine was utilized as a positive control due to the low expression of RIPK3 in OS, hFOB, and hMSC cell lines. Western blot confirmed absence of protein expression in these OS cell lines, where G292 cells transfected with RIPK3 were used as a control (Figure 2B). MLKL expression was also found to be significantly reduced in human OS cell lines compared to hMSC and hFOB normal cell lines (Figure 2C). Together, these data demonstrate that aberrant methylation inhibits RIPK3 transcription and translation in OS cell lines.

### 2.3. RIPK3 Expression in OS Cells Lines Does Not Alter Survival Without Therapy but Significantly Increases Susceptibility to Cisplatin Through Activation of Necroptosis

**Figure 3:**
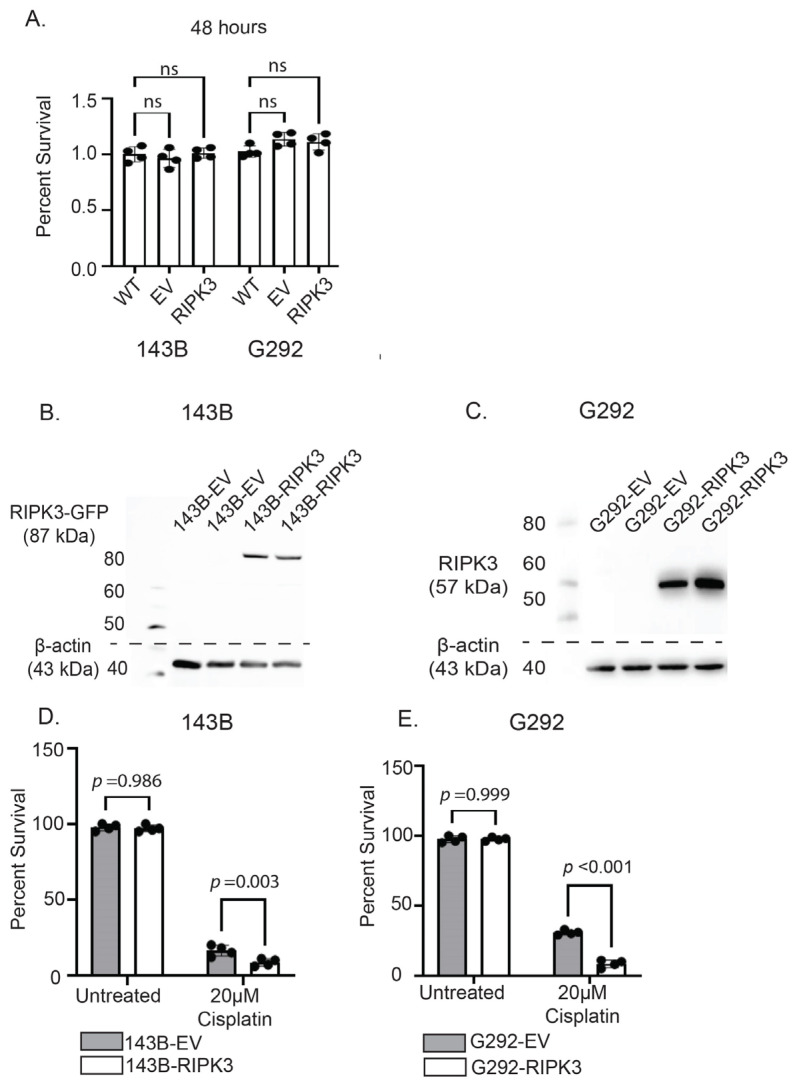
Forced expression of RIPK3 augments cisplatin sensitivity in 143B and G292 cell lines. (**A**) Enforced expression of RIPK3 in 143B and G292 cell lines does not alter overall survival. (**B**) Western blot confirms forced expression of RIPK3 in stably transduced 143B cells (GFP-tagged RIPK3; 87 kDa). (**C**) Western blot confirms forced expression of RIPK3 in stably transfected G292 cells (RIPK3; 57 kDa). (**D**) 143B cells expressing RIPK3 (143B-RIPK3) demonstrate reduced survival following 48 h cisplatin treatment compared to empty vector (EV) and untreated controls. (**E**) G292 cells expressing RIPK3 (G292-RIPK3) are more susceptible to cisplatin-induced cell death compared to EV and untreated controls. ns = not significant.

With decreased RIPK3 expression associated with aberrant CpG island methylation observed in OS primary tumors and cell lines, we first investigated the effect of enforced RIPK3 on 143B and G292 cell viability. At 48 h (Figure 3A), no significant differences were seen between cells with RIPK3 overexpression compared to empty vector (EV) or no treatment (WT) controls. Next, we examined the effects of RIPK3 forced expression on susceptibility to cisplatin chemotherapy. OS cell lines 143B and G292 were transduced and transfected, respectively, with RIPK3 and stably transduced/transfected cell lines were selected that exhibited RIPK3 protein expression by Western blot analysis (Figure 3B,C). Empty vector cell lines (EV) were transduced/transfected without the RIPK3 gene and used as controls.

RIPK3-expressing 143B cells were then subjected to treatment with cisplatin (20µM) for 48 h, and survival was assessed compared to EV and untreated controls. 143B cells expressing RIPK3 demonstrated a significant reduction in survival compared to EV and untreated controls (Figure 3D). This correlation was also observed in G292-RIPK3 cells (Figure 3E). A dose–response relationship in cell survival was seen in both the 143B-RIPK3 and G292-RIPK3 cell lines, with higher doses of cisplatin correlating to lower rates of survival compared to controls (Figure S1). The reduction in survival was more pronounced in cells expressing RIPK3. These results confirmed increased cytotoxicity with cisplatin exposure in OS cells with forced expression of RIPK3.

### 2.4. Knockdown of RIPK3 Expression Desensitizes OS Cells to Cisplatin Cytotoxicity

**Figure 4:**
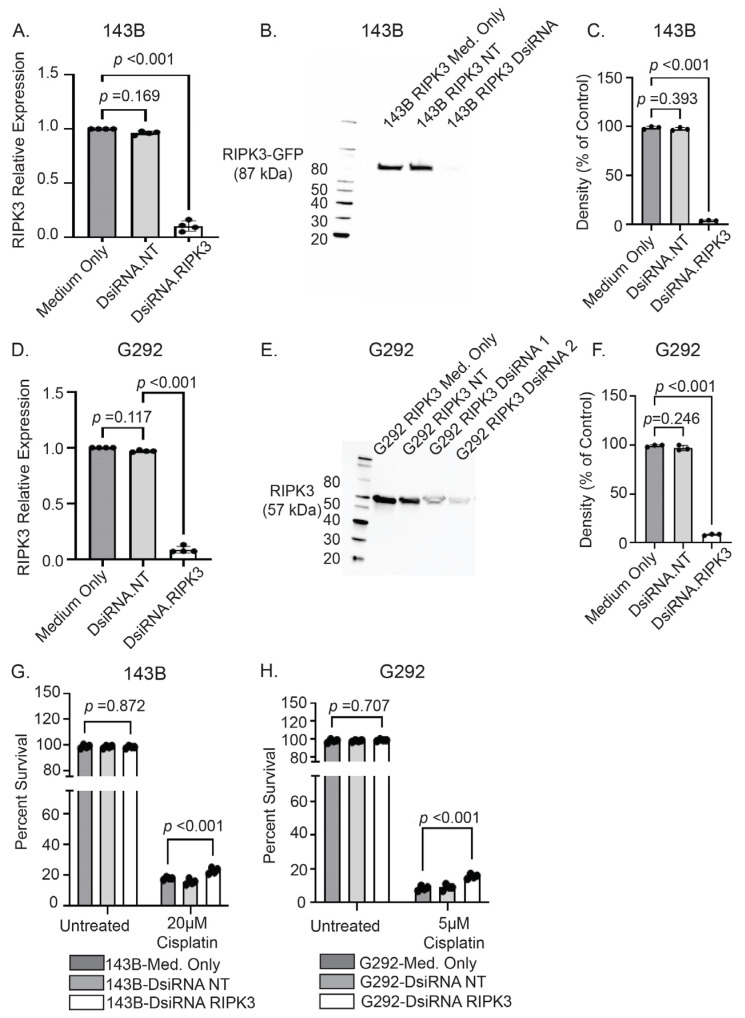
DsiRNA silencing of *RIPK3* restores cisplatin chemoresistance. (**A**) DsiRNA treatment successfully reduced *RIPK3* mRNA and (**B**,**C**) protein levels in 143B cells with stable RIPK3 expression. Similarly, DsiRNA treatment reduced RIPK3 (**D**) mRNA and protein levels (**E**,**F**) in RIPK3 expressing G292 (1: 50 nM DsiRNA, 2: 75 nM DsiRNA). Following 48 h of cisplatin treatment (20 µM), (**G**) 143B and (**H**) G292 cells with DsiRNA treatment to reduce RIPK3 expression demonstrated increased chemoresistance compared to untreated and non-targeting (NT) controls.

To determine whether loss of RIPK3 expression would abrogate the cisplatin chemosensitivity acquired by RIPK3 forced expression, we utilized dicer short substrate interfering RNAs (DsiRNA) to knockdown RIPK3 protein expression in these cell lines [43]. In 143B cells expressing RIPK3, *RIPK3* mRNA levels were significantly reduced following treatment with *RIPK3*-targeting DsiRNA (Figure 4A). This was further validated with Western blot, which demonstrated a significant reduction in RIPK3 protein in DsiRNA-*RIPK3*-treated cells compared to non-targeting or untreated controls (Figure 4B,C). Additionally, *RIPK3* mRNA (Figure 4D) and RIPK3 protein (Figure 4E,F) levels were significantly reduced in G292 cells stably expressing RIPK3 following DsiRNA treatment. When exposed to cisplatin (48 h at 20 µM), both the 143B (Figure 4G) and G292 (48 h at 5 µM due to enhanced cisplatin sensitivity) (Figure 4H) cell lines demonstrated decreased survival following treatment with cisplatin, but those with *RIPK3* silencing demonstrated the highest chemoresistance compared to untreated and non-targeting controls. This result indicated that reduction in *RIPK3* restored relative cisplatin chemoresistance in human OS cells.

### 2.5. RIPK3 Expression in OS Cells Is Associated with Activation of Molecular Effectors of Necroptosis Following Cisplatin Treatment

**Figure 5:**
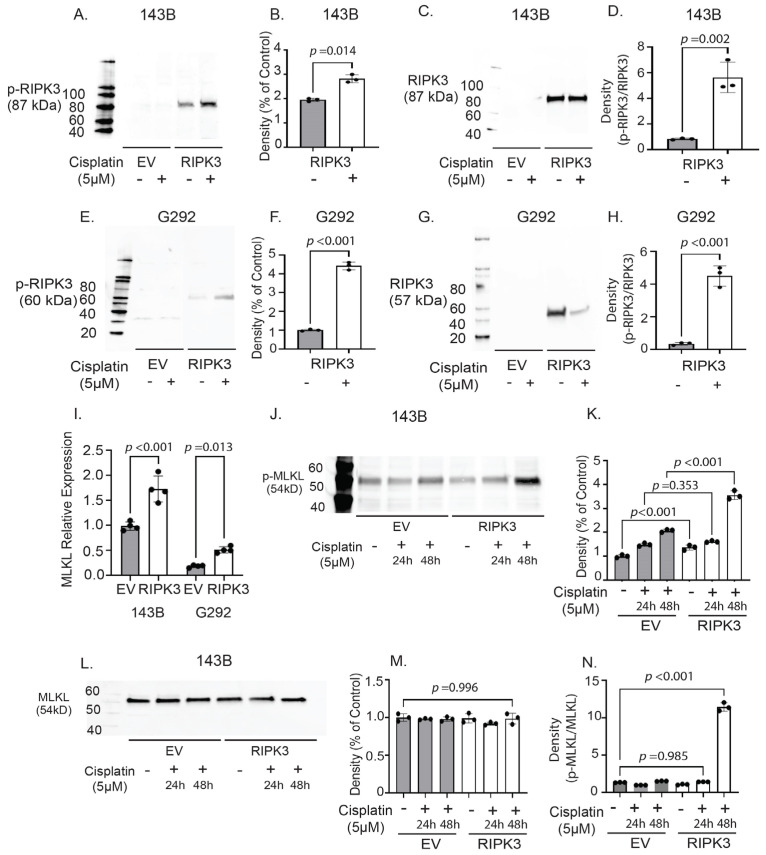
Cisplatin treatment results in phosphorylation of RIPK3 and activation of MLKL-associated necroptosis. (**A**) 143B cells stably expressing RIPK3 demonstrate elevated levels of phosphorylated RIPK3 (p-RIPK3; 87 kDa) following cisplatin treatment compared to EV controls. (**B**) Quantification of 143B p-RIPK3 Western blot densitometry with untreated as control. (**C**) We compared expression of p-RIPK3 to total protein by Westerbn blot and (**D**) quantified changes following RIPK3 forced expression. (**E**) RIPK3 expressing G292 cells also show a significant increase in p-RIPK3 (60 kDa) levels compared to EV and untreated controls. (**F**) Quantification of G292 p-RIPK3 Western blot densitometry with untreated RIPK3 as control. (**G**) We compared changes in p-RIPK3 to total protein changes by Western blot and (**H**) through densitometry analysis. (**I**) *RIPK3* mRNA expression is elevated in RIPK3 expressing 143B and G292 cells. (**J**) 143B cells stably expressing RIPK3 show significantly elevated levels of phosphorylated MLKL (p-MLKL; 54 kDa) following both 24 and 48 h cisplatin treatment compared to EV and untreated controls. (**K**) Quantification of 143B p-MLKL Western blot densitometry with untreated EV as control. (**L**) Examination of total MLKL protein levels by Western blot and (**M**) densitometry. (**N**) Finally, we examined expression levels of p-MLKL/total MLKL and demonstrate that forced expression of RIPK3 leads to an activation of necroptosis via p-MLKL.

Noting decreased survival in cells expressing RIPK3, we wished to confirm that RIPK3 was activating the necroptosis cell death pathway. Upon activation, RIPK3 is phosphorylated to upregulate necroptosis. Phospho-RIPK3 (p-RIPK3) is a known activator of the mixed lineage kinase (MLKL), the terminal effector protein for necroptosis [36,44,45]. We compared p-RIPK3 protein levels by Western blot in OS cells with and without RIPK3 expression after 48 h of culture in medium with or without cisplatin. Comparing cisplatin-treated 143B-RIPK3 cell lines to EV and untreated cells and RIPK3 total protein (Figure 5C), we observed increased phosphorylation of RIPK3 (p-RIPK3) (Figure 5A) and quantified these results (Figure 5D). In contrast, EV controls, which lack RIPK3 expression, demonstrated no change in p-RIPK3 levels with cisplatin treatment (Figure 5A,B,D). To further confirm these findings, we examined p-RIPK3 levels in G292 cells with EV and those stably expressing RIPK3 (Figure 3B) compared to RIPK3 total protein (Figure 5G). Following cisplatin treatment, the G292-RIPK3 cells demonstrated a significant increase in p-RIPK3 compared to both untreated and EV controls (Figure 5F,H).

We next assayed phospho-MLKL (p-MLKL) expression with and without cisplatin treatment in these cell populations. First, we examined *MLKL* mRNA expression via qRT-PCR and found that the cell lines stably expressing *RIPK3* demonstrated significantly higher expression of *MLKL* (Figure 5I). When we examined the levels of p-MLKL in 143B cells expressing RIPK3 (143B-RIPK3), we detected significant increases in the levels of p-MLKL compared to cisplatin-untreated and EV controls (Figure 5J,K) and compared to total MLKL (Figure 5L). Elevation of the necroptosis molecular effectors p-RIPK3 and p-MLKL following cisplatin exposure is consistent with increased activation of necroptosis, demonstrating that in cell lines with enforced expression of this tumor suppressor that are exposed to chemotherapeutic agents, there was increased activation of this cell death pathway (Figure 5M,N) [35,46].

## 3. Discussion

Treatment of localized OS with surgery and neoadjuvant and adjuvant chemotherapy results in long term survival in approximately 65% of cases [47,48]. The introduction of the high-dose methotrexate, doxorubicin, cisplatin (MAP) regimen in the 1980s resulted in markedly improved survival with decreased metastatic failure compared to treatment with surgery alone in these localized cases, presumably reflecting effective eradication of radiographically undetectable metastatic disease present at the time of surgery [4,49]. The necessity of effective resection of the primary tumor for optimal disease control is demonstrated by the inferior survival outcomes observed in cases with unresectable primary tumor sites such as axial (e.g., pelvic or vertebral) primary lesions [50,51,52]. Bulk residual primary tumor is poorly responsive to chemotherapy, whether due to reduced drug penetration into tumor tissue or innate cellular chemoresistance. Likewise, multifocal tumors and cases with radiographically evident metastatic disease at the time of diagnosis exhibit poor chemotherapeutic response and have very poor treatment outcomes [13,53]. Despite decades of research, effective chemotherapies to salvage MAP-resistant tumors have not been identified.

Much research has focused on defining the molecular determinants of chemoresistance in OS. Examinations of drug transport efficiencies demonstrate that OS cells express reduced levels of folate carrier transporters, which confers resistance to methotrexate via reduced intercellular transport [54,55]. Elevated levels of apurinic endonuclease (APE-1), a key enzyme in DNA base excision repair, have been detected in OS patients and correlate with chemo resistance, decreased survival and increased possibility of relapse [56,57]. Abnormal levels of matrix metalloproteinases two, three, and nine *(MMP-2*, *MMP-3*, *MMP-9*) have also been shown to contribute to doxorubicin and cisplatin resistance, respectively, in OS [58,59,60]. Mutations in genes associated with the WNT, PI3K, MAPK, and mTOR pathways in OS tumors have also been shown to contribute to chemo resistance [61,62,63,64]. Upregulation of high mobility group box 1 protein (HMGB1) has been shown to confer resistance by initiating a cytoprotective autophagic response [65]. Similarly, elevated levels of hypoxia inducible factor 1-α (HIF1-α) contributes to both a hypoxic tumor microenvironment and chemo resistance [66,67,68]. Recent examinations have also shown that various ncRNAs (including microRNAs, circular RNAs, and long non-coding RNAs) also regulate cellular responses governing OS chemo resistance [69,70,71,72,73]. Finally, genome-wide DNA methylation profiling found correlations between increased methylation at loci and incidence of relapse [74]. In our analysis, restricted to CpG islands, a disproportionate gain of aberrant methylation compared to nonmalignant controls was identified in less than 25% of potential targets. Among the gene-associated CpG islands exhibiting the highest aberrant methylation, we identified the RIPK3 CpG island.

RIPK3 has been shown to activate and regulate the necroptosis programmed cell death pathway in OS and other cancers [13,37,54,75]. In the pathology of cancer, genetic and/or epigenetic events result in disruption of normal programmed cell death feedback signaling and contribute to drug resistance [14,76]. Activation of RIPK3 can occur via multiple signals including TNF-R1, CD95, death receptors, toll-like receptors, and Z-DNA binding protein 1 [77]. After auto-phosphorylation, RIPK3 then phosphorylates mixed lineage kinase domain protein (MLKL) to induce MLKL translocation and loss of membrane integrity, ultimately leading to membrane permeabilization and cell death [78,79]. Our results suggest that aberrant DNA methylation-associated silencing of *RIPK3* expression impairs the activation of necroptotic cell death following cisplatin exposure in OS cells. While classic necroptosis requires RIPK3 phosphorylation, recent studies have determined that MLKL can regulate cell death in an RIPK3-independent manner in non-RIPK3 expressing cells, including OS cell lines [80,81]. Nevertheless, our results in OS cell lines indicated that acquired RIPK3 expression augmented MLKL phosphorylation and cell death following cisplatin exposure, suggesting that *RIPK3* silencing conferred relative resistance to cisplatin cytotoxicity despite intact MLKL expression.

Recent scientific and genetic advances in pediatric oncology have begun to uncover unique genetic mutations and molecular mechanisms underlying chemotherapeutic resistance. For example, genomic studies of OS tumors have identified OS-specific silencing translocations and have begun to elucidate the role of infiltrating immune cells in the OS tumor microenvironment [82,83,84]. These findings are paving the way for the development of novel drug delivery mechanisms and immuno- and pharmacotherapies for a range of cancer types. For instance, nanotechnologies such as lipid-based or polymer-based nanoparticles are being evaluated to improve therapeutic efficacy in pediatric central nervous system malignancies, leukemia, lymphoma, and bone cancers [85,86]. While mutational differences between adult- and pediatric-onset cancers have been shown to limit the efficacy of some available immunotherapies, CAR T-cell therapy has proven an effective adjuvant therapy in the treatment pediatric B-cell acute lymphoblastic leukemia [87]. Epigenetic therapy inhibiting histone deacetylases (HDACi), often upregulated in many cancers, has also shown promising preclinical findings in the treatment of pediatric neuroblastoma, leukemia, and sarcomas [88,89,90]. In adults, therapies such as photoactivated DNA nanodrugs, miRNA mimics, and DNA methyltransferase inhibitors have shown promise in reversing chemotherapeutic resistance in solid tumors [91,92,93]. These approaches may also demonstrate efficacy in OS, once appropriate targets are identified. Towards this end, optimization of pharmacologic therapy to effect RIPK3 demethylation may represent a novel therapeutic intervention that facilitates improved outcomes in pediatric patients with chemo resistant osteosarcoma.

## 4. Materials and Methods

### 4.1. Cell Lines and Culture Conditions

Human osteosarcoma (OS) cell lines HOS (CRL-1543), 143B (CRL-8303), and G292 (CRL-1423) and human fetal osteoblasts (hFOB; CRL-3602) were obtained from the American Tissue Culture Collection (ATCC, Manassas, VA, USA). Human mesenchymal stem cells (hMSC; PT-2501) were obtained from Lonza (Basel, Switzerland). The OS cell lines were grown in Eagle’s Minimum Essential Medium (Thermo Fisher; MT10009CV, Waltham, MA, USA) supplemented with 10% fetal bovine serum (E10 medium), hMSCs in the MSCGM Mesenchymal Stem Cell Growth Medium BulletKit (Lonza; PT-3001, Basel, Switzerland), and hFOBs in 1:1 Ham’s F12/Dulbecco’s Minimum Essential Medium without phenol red (Sigma Aldrich; D6434, St. Louis, MO, USA), with 2.5 mM L-glutamine, 0.3 mg/mL G418, and 10% FBS. All cells were maintained in a humified incubator at 37 °C with 5% CO_2_.

### 4.2. Human Primary OS Tumor Samples

All human tissue samples were obtained under approved Akron Children’s Hospital IRB regulations (protocol #1055849) and were consent-exempt based on the use of “to be discarded” tissue for a non-clinical study under 45 CFR. Primary human OS tumor samples were obtained from post-chemotherapy resections performed at Akron Children’s Hospital (Akron, OH, USA). The resections were decalcified for ~3 weeks in EDTA, and the percentage of viable tumor was determined by microscopic evaluation by a pediatric pathologist. Samples were cut into 0.2–0.3 g pieces and stored in liquid nitrogen until DNA extraction.

### 4.3. DNA Isolation

DNA was prepared from OS cell lines, hFOBs, and hMSCs using the Puregene Blood Core Kit following the manufacturer recommended protocols (Qiagen; 158026 Hilden, Germany). For the primary tumor samples, tumor fragments were ground with a mortar and pestle over liquid nitrogen and DNA was prepared with the ReliaPrep gDNA Tissue Miniprep System following recommended protocols (Promega; A2051, Madison, WI, USA) [94]. DNA from normal human bone was prepared from surgical specimens of supernumerary digits. DNA concentration and quality were assessed using a Nanodrop^®^ 2000c spectrophotometer (Thermo Fisher, Waltham, MA, USA).

### 4.4. Methylation Profiling and Analysis

Methylation profiling was performed with the Illumina Infinium HumanMethylation450 BeadChip Assay at the Lerner Research Institute (Cleveland Clinic, Cleveland, OH, USA). Samples included DNA from 16 primary human osteosarcomas, 2 non-malignant human bone samples, 4 human normal tissue samples (adult skeletal muscle, fetal liver, adult adipose, and fetal brain (BioChain Institute, Newark, CA, USA)), and 2 cultured hMSC samples. Data were reported as diff scores (D) that reflected the prevalence of methylation for each tumor sample compared to pooled controls. D is reported for each of the >480K individual CpG sites. A higher D signifies a higher prevalence of methylation in the tumor sample relative to controls. No statistical test was utilized to determine D because there is no specific level of aberrant CpG island methylation associated with transcriptional silencing. More methylation just means a greater likelihood of silencing. We chose a conservatively but not restrictively high level for D (300) to insure that CpG islands identified by our analysis were likely to be silenced.

We restricted the analysis to CpG island-associated CpG sites and rank ordered CpG islands on an Observed/Expected (Obs/Exp) continuum, where Obs = the number of CpG site “methylation hits” in the CpG island; a “hit” is a CpG site where the reported D is greater than a threshold D selected for the analysis (|D| > |D_Th_|); and Exp = the number of CpG sites in the island interrogated by the assay multiplied by the total number of “methylation hits” in the assay/the total number of CpG sites interrogated by the assay. Thus, Exp = # CpG sites in the CpG island multiplied by the total # hits in the assay/485,578. CpG islands exhibiting the highest Obs/Exp values were candidates for further evaluation.

### 4.5. Transfections

The *RIPK3* coding sequence was amplified from the pCMV-SPORT6-RIPK3 vector (TransOMIC Technologies; TCH1303, Huntsville, AL, USA) with primers (F: 5′ GCT AGC TGG CAC CTT CCA GCC TGA TG 3′ and R: 5′ AAG CTT TTA TTT CCC GCT ATG ATT ATA CC 3′) to include a Kozak consensus sequence and NheI and HindIII restriction sites for cloning. The purified PCR product was then cloned into pCR2.1-TOPO vector following recommended protocols (Thermo Fisher; 451641, Waltham, MA, USA) [95,96]. The pCR2.1-RIPK3 and the mammalian expression vector pcDNA3.1Neo (Thermo Fisher; V790-20,Waltham, MA, USA) were digested using NheI and HindIII restriction enzymes (New England BioLabs, Ipswich, MA, USA). The RIPK3 fragment and the digested pcDNA3.1Neo were purified with the Zymoclean Gel DNA Recovery Kit and the DNA Clean & Concentrator-5 kit, respectively (Zymo Research; D4001 and D4003, Irvine, CA, USA). A ligation reaction was performed with T4 DNA Ligase, and reactions were transformed into Top10 competent cells (Thermo Fisher, Waltham, MA, USA). The RIPK3 sequence was confirmed by DNA sequencing (Functional Biosciences, Madison, WI, USA). The OS cell line G292 was stably transfected with either pcDNA3.1Neo (control vector) or pcDNA3.1Neo-RIPK3 using FuGENE 6 transfection reagent (Promega; E2691, Madison, WI, USA). Colonies were grown with selection agent aminoglycoside antibiotic G418 at either 200 µM or 400 µM (Sigma Aldrich; 108321-42-2, St. Louis, MO, USA). Colonies were selected, expanded, and screened for RIPK3 expression with Western blot.

### 4.6. Transductions

Lentiviral particles containing the pLenti-C-mGFP-P2A-Puro control vector (PS100093V5) and particles containing pLenti-C-mGFP-P2A-Puro-RIPK3 ORF (RC209549L4V) were purchased from Origene (Rockville, MD, USA) and transduced into the OS cell line 143B using an MOI of 0.25 and 8 µg/mL polybrene. Colonies were selected by being grown in 1 µg/mL puromycin and then expanded and screened for RIPK3 expression with Western blot.

### 4.7. Pyrosequencing

Genomic DNA was submitted to EpigenDx, Inc. (Hopkinton, MA, USA) for bisulfite modification and subsequent pyrosequencing [94]. In brief, 500 ng of extracted genomic DNA was bisulfite-treated using the EZ DNA Methylation kit (Zymo Research, Inc., CA). Bisulfite-treated DNA was purified according to the manufacturer’s protocol and eluted to a final volume of 46 μL. Next, PCRs were performed using 1 μL of bisulfite-treated DNA and 0.2 μM of each primer. One primer was biotin-labeled and HPLC purified in order to purify the final PCR product using sepharose beads. PCR product was bound to Streptavidin Sepharose HP (GE Healthcare Life Sciences, Chicago, IL, USA), after which the immobilized PCR products were purified, washed, denatured with a 0.2 μM NaOH solution, and rewashed using the Pyrosequencing Vacuum Prep Tool (Pyrosequencing, Qiagen), as per the manufacturer’s protocol. Sequencing primer, 0.5 μM, was then annealed to the purified single stranded PCR products. PCR products were sequenced by Pyrosequencing on the PSQ96 HS System (Pyrosequencing, Qiagen) following the manufacturer’s instructions.

The methylation status of each CpG site was determined individually as an artificial C/T SNP using QCpG software (Pyrosequencing, Qiagen). The methylation level at each CpG site was calculated as the percentage of the methylated alleles divided by the sum of all methylated and unmethylated alleles. The mean methylation level was calculated using methylation levels of all measured CpG sites within the targeted region of each gene. Each experiment included non-CpG cytosines as internal controls to detect incomplete bisulfite conversion of the input DNA. In addition, a series of unmethylated and methylated DNA are included as controls in each PCR. Furthermore, PCR bias testing was performed by mixing unmethylated control DNA with in vitro methylated DNA at different ratios (0%, 5%, 10%, 25%, 50%, 75%, and 100%), followed by bisulfite modification, PCR, and Pyrosequencing analysis. The methylation status of normal bone (n = 2), human mesenchymal stem cells (n = 1), human fetal osteoblasts (n = 1), OS cell lines (n = 5), and OS primary tumors (n = 11) was probed across six CpGs in the RIPK3 gene (ASY1409). The six CpGs are located 304 to 346 base pairs from the transcriptional start site on Chromosome 14: 24339742-24339700. Methylation and negative controls were also run.

### 4.8. Next Generation Sequencing

Next generation sequencing analysis was performed by EpigenDx on the same 20 genomic DNA samples to measure the percent methylation of CpGs in MLKL and RIPK3. Twelve CpGs across MLKL (Assays ASY4109, ASY4134, and ASY4154) and nine in RIPK3 (Assay ASY1409) were interrogated. Methylation and negative controls were also included. In brief, 500 ng of extracted DNA samples were bisulfite modified using the EZ-96 DNA Methylation Kit™ (ZymoResearch; D5004, Irvine, CA, USA), per the manufacturer’s protocol with minor modification. The bisulfite-modified DNA samples were eluted using M-elution buffer in 46 μL. Then, bisulfite-modified DNA samples were amplified using separate multiplex or simplex PCRs. PCRs included 0.5 units of HotStarTaq (Qiagen; 203205, Hilden, Germany), 0.2 μM primers, and 3 μL of bisulfite-treated DNA in a 20 μL reaction. All PCR products were verified using the Qiagen QIAxcel Advanced System (v1.0.6). Prior to library preparation, PCR products from the same sample were pooled and then purified using the QIAquick PCR Purification Kit columns or plates (Qiagen; 28106 or 28183, Hilden, Germany). Libraries were prepared using a custom Library Preparation method created by EpigenDx. Next, library molecules were purified using Agencourt AMPure XP beads (Beckman Coulter; A63882, Brea, CA, USA). Template preparation and enrichment were performed on the Ion Chef™ system using Ion 520™ and Ion 530™ ExT Chef reagents (Thermo Fisher; A30670, Waltham, MA, USA). Following this, enriched, template-positive library molecules were sequenced on the Ion S5™ sequencer using an Ion 530™ sequencing chip (A27764).

FASTQ files from the Ion Torrent S5 server were aligned to a local reference database using the open-source Bismark Bisulfite Read Mapper program (v0.12.2) with the Bowtie2 alignment algorithm (v2.2.3). Methylation levels were calculated in Bismark by dividing the number of methylated reads by the total number of reads. An R-squared value (RSQ) was calculated from the controls set at known methylation levels to test for PCR bias.

### 4.9. Western Blots

Protein was harvested from cultured cells after lysis in Radioimmunoprecipitation (RIPA) buffer with protease and phosphatase inhibitor [94,97]. Aliquots of 30 µg were separated on mini-PROTEAN 4–15% TGX Stain-Free gels (Bio-Rad Laboratories, Hercules, CA, USA) and transferred to low fluorescence polyvinylidene difluoride (LF-PVDF) membrane. Membranes were blocked in 5% nonfat dry milk or bovine serum albumin (BSA) and then incubated overnight in primary antibody followed by secondary antibody incubation for one hour. Primary antibodies include RIPK3 (Origene; TA80311,Rockville, MD, USA, 1:750), phospho-RIPK3 (Abcam; ab209384, Waltham, MA, USA;, 1:1000), and phospho-MLKL (Origene; TA378561, Rockville, MD, USA, 1:1000). Secondary antibodies include goat anti-rabbit HRP (Thermo Fisher; 31460, Waltham, MA, USA;, 1:5000) and goat anti-mouse (Thermo Fisher; 31430, Waltham, MA, USA;, 1:5000). Western blot analyses were completed using stain-free imaging, where total protein normalization was utilized in place of housekeeping load controls [98,99,100] (Sule, Gurtler, Gilda). Imaging was accomplished using the ChemiDoc MP system in combination with Image Lab v6.1 software (Bio-Rad Laboratories, Hercules, CA, USA). Untouched and unmodified Western Blots are presented in Figure S2.

### 4.10. RT-qPCR

Total RNA was prepared from cultured cells with the RNeasy Mini Kit, following manufacturer recommended protocols (Qiagen; 74104, Hilden, Germany). cDNA was synthesized using the Protoscript II First Strand cDNA Synthesis Kit (New England Biolabs; 6560, Ipswich, MA, USA). Quantitative PCR was performed in triplicate using the PrimeTime qPCR Assays (Integrated DNA Technologies, Coralville, IA, USA) for RIPK3 (Hs.PT.58.1390703.g) and MLKL (Hs.PT.58.14577320). PrimeTime qPCR Assay for Hypoxanthine Phosphoribosyltransferase 1 (HPRT1) was utilized as control (Hs.PT.58v.45621572) [101,102]. All reactions were conducted on an Agilent Technologies Stratagene Mx3005P machine.

### 4.11. RIPK3 DsiRNA Knockdown

Three dicer-substrate short interfering RNAs (DsiRNAs) against RIPK3 and a non-targeting control DsiRNA were purchased from Integrated DNA Technologies (Coralville; hs.Ri.RIPK3.13, IA, USA;). Cells were seeded at a density of 6 × 10^5^/well in 6-well tissue culture plates the day before transfection and allowed to adhere overnight. The cells were transfected with Lipofectamine RNAiMAX (Thermo Fisher; 13778100, Waltham, MA, USA) in Opti-MEM, with a final concentration of 25 nM DsiRNA. Each of the three DsiRNAs were transfected individually and pooled. At 24 h post transfection, medium/complexes were replaced with complete medium. At 48 h post transfection, RNA and protein were harvested to confirm the RIPK3 knockdown, and cells were then seeded in 96 well plates for cisplatin sensitivity assays.

### 4.12. Cisplatin Sensitivity Assay

Cells were seeded at 1 × 10^4^ cells per well into 96-well tissue culture plates in E10 complete medium without selective agent. The next day, cells were treated with a range of cisplatin (Accord Healthcare, Durham, NC, USA) concentrations in quadruplicate with experiment-specific concentrations described in text. Forty-eight hours after the addition of cisplatin, percent survival was determined using the CellTiter 96 Aqueous One Solution Reagent (MTS; Promega; G3582, Madison, WI, USA), following recommended protocols. In brief, 20 µL of MTS solution was added to a 96-well containing 100 µL of cell culture medium. Wells were incubated at 37 °C with 5% CO_2_ for two hours and absorbance at 490 nm was read on a Synergy H1 plate reader (Agilent, Santa Clara, CA, USA). Percent survival was calculated relative to untreated controls.

### 4.13. Statistical Analyses

Data were analyzed using GraphPad Prism v10.4.1 software (GraphPad, San Diego, CA, USA). All experiments utilized either three or four replicates (details in text) per experiment. Data were analyzed using one-way Anova with Tukey’s post hoc tests for multiple-group analyses. Two-group analyses used unpaired *t*-tests. Results were determined to be parametric due to similar OS stage, use of validated cell line replicates, and the use of relevant sample sizes for all experiments based on standard laboratory practices in the field [103,104,105,106]. Results were considered significant when *p*-values were less than 0.05. Data were graphed as mean(s) ± standard error of the mean (SEM).

## Figures and Tables

**Table 1:** Percent mean methylation identified in OS cell lines, healthy control samples, and OS tumor samples resected following induction chemotherapy. Methylation was assessed by pyrosequencing and next generation sequencing (NGS). Samples analyzed included several cell types (human mesenchymal stem cells (hMSC), human fetal osteoblasts (hFOB)), OS cell lines (HOS, G292, MNNGHOS, MG63, 143B), two non-cancerous bone samples, OS tumor samples (n = 11), and pyrosequencing or NGS controls.

	Mean Percent Methylation—RIPK3	
Sample	Pyrosequencing	NGS
hMSC	10.6	12.1
hFOB	18.9	20.1
HOS	62.9	78.7
G292	87.9	95.7
MNNGHOS	88.7	94.9
MG63	91.0	96.2
143B	88.9	94.7
Non-cancerous bone 1	3.6	1.4
Non-cancerous bone 2	1.0	0.7
OS 1	3.2	2.6
OS 2	4.6	4.4
OS 3	0.6	4.5
OS 4	31.4	46.0
OS 5	34.4	54.5
OS 6	1.5	-
OS 7	44.4	55.6
OS 8	6.7	8.0
OS 9	45.6	56.7
OS 10	26.4	30.4
OS 11	33.1	44.5

## Data Availability

The data presented in this study are available on request from the corresponding author. The data are not publicly available due to ongoing studies.

## Supplementary Materials

The supporting information can be downloaded at: https://www.mdpi.com/article/10.3390/ijms26083863/s1.

## Abbreviations

The following abbreviations are used in this manuscript:

OS	Osteosarcoma
RIPK3	Receptor interacting protein kinase-3
IPF	Idiopathic pulmonary fibrosis
MS	Multiple sclerosis
ALS	Amyotrophic lateral sclerosis
AD	Alzheimer’s disease
DsiRNA	Dicer substrate RNA
MLKL	Mixed lineage kinase domain-like
hFOB	Human fetal osteoblasts
hMSC	Human mesenchymal stem cells
