# Supplementary material for: Epigenetic Inactivation of RIPK3-Dependent Necroptosis Augments Cisplatin Chemoresistance in Human Osteosarcoma
Source: Int J Mol Sci. 2025 Apr 18;26(8):3863. doi: 10.3390/ijms26083863 (PMC12027565; doi:10.3390/ijms26083863)
Supplement: Supplementary file 1 [file ijms-26-03863-s001.zip › ijms-3502998-supplementary.pdf]

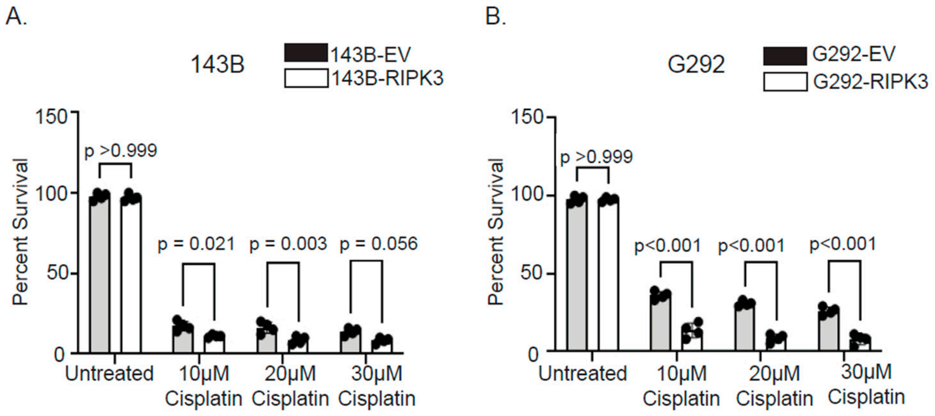

Figure S1: Cisplatin treatment in RIPK3 expressing cells demonstrate dose-dependent survival.

Figure S2: Original and unmodified Western Blot images:

Supplemental Figure 2: Untouched and stain-free Western blot images:

Figure 2B: Western blot analysis demonstrates RIPK3 expression is absent in OS cell lines HOS, 143B, MG63, G292 and MNNG/HOS, in hMSCs, and hFOBs. G292 RIPK3 transfected cells were used as a positive control. RIPK3, 57 kDa.

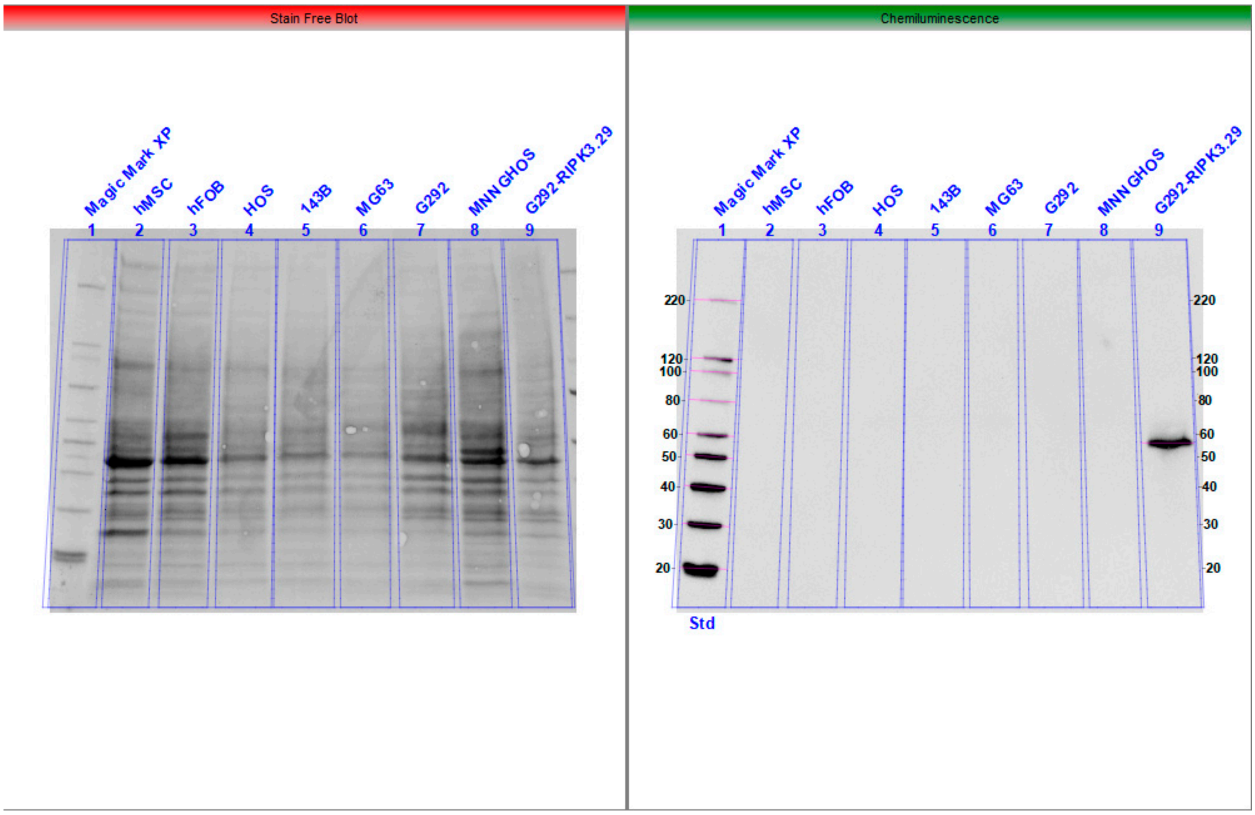

Figure 3B: Western blot confirms forced expression of RIPK3 in stably transduced 143B cells (GFP-tagged RIPK3; 87kDa). Bands within the green box used in manuscript figure.

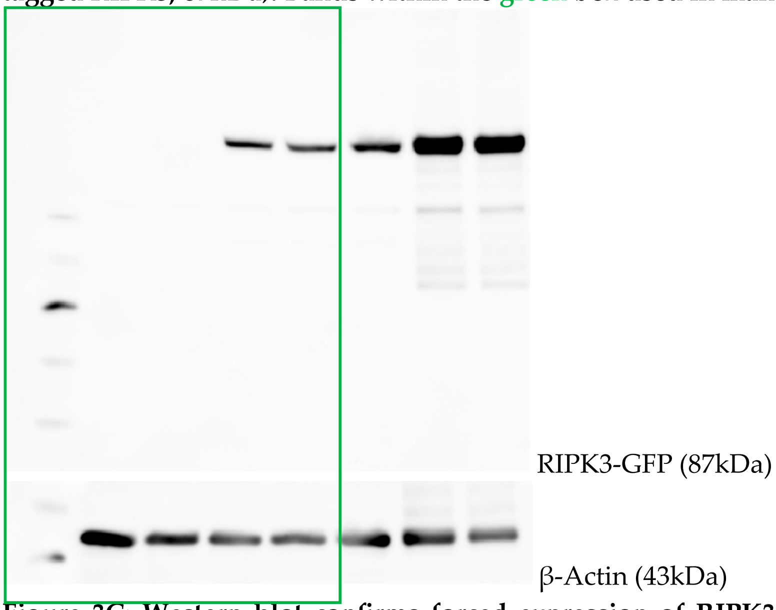

Figure 3C: Western blot confirms forced expression of RIPK3 in stably transfected G292 cells (RIPK3; 57kDa). Bands within the green box used in manuscript figure.

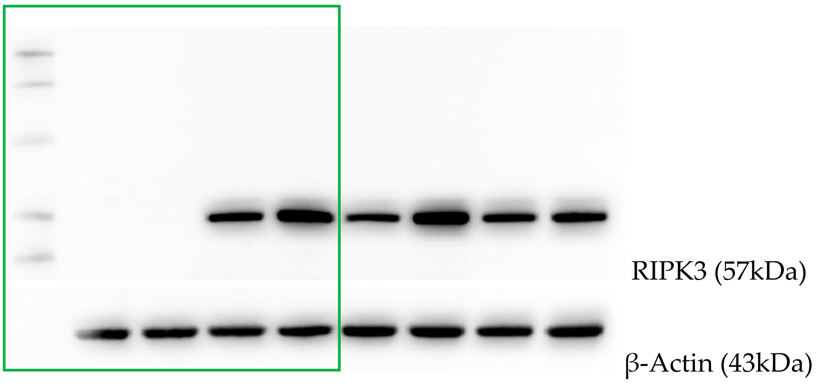

Figure 4B: DsiRNA treatment successfully reduced RIPK3 mRNA and (B, C) protein levels in 143B cells with stable RIPK3 expression. Gel image was reflected horizontally. Bands within the green box used in manuscript figure. RIPK3 87 kDa.

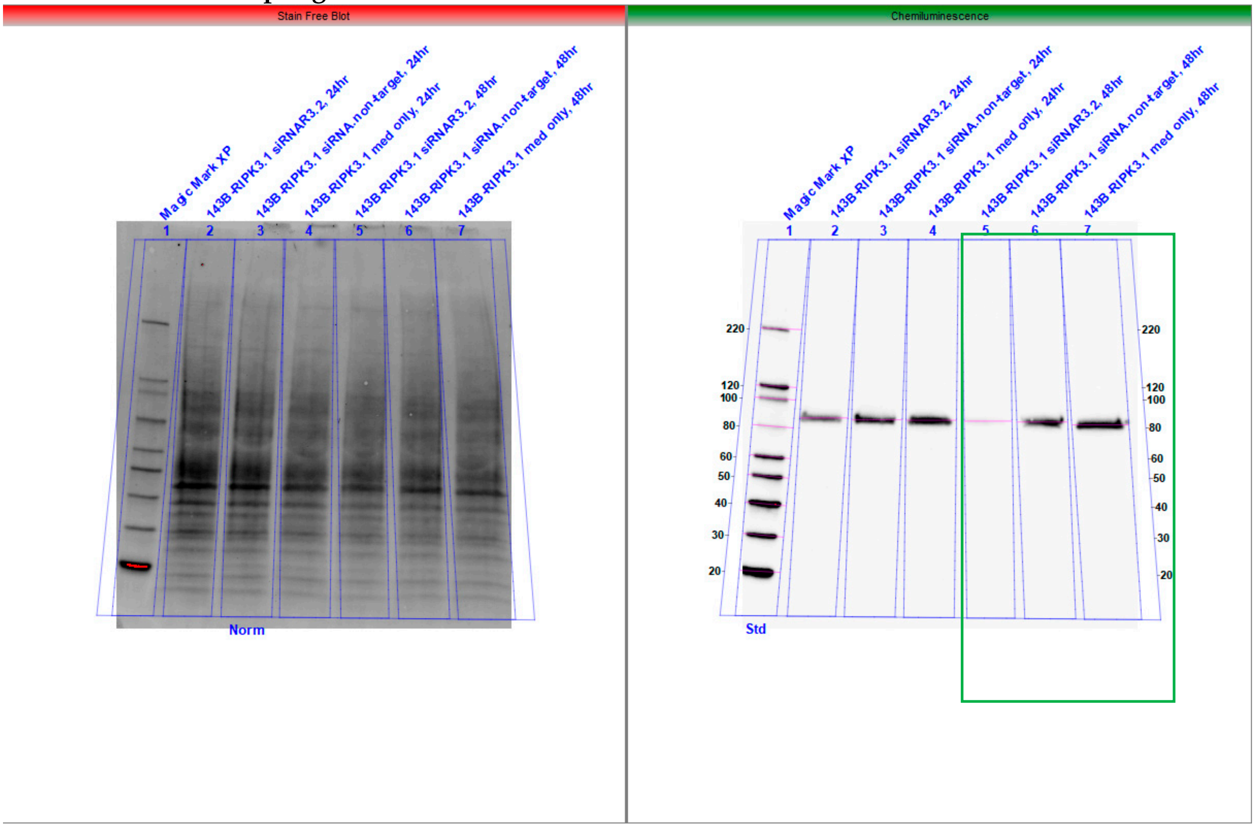

Figure 4E: Similarly, DsiRNA treatment reduced RIPK3 C) mRNA and protein levels (E, F) in RIPK3 expressing G292. RIPK3 57 kDa. Bands within the green box used in manuscript figure (with 50nM DsiRNA labeled as "1" and 75nM DsiRNA labeled as "2").

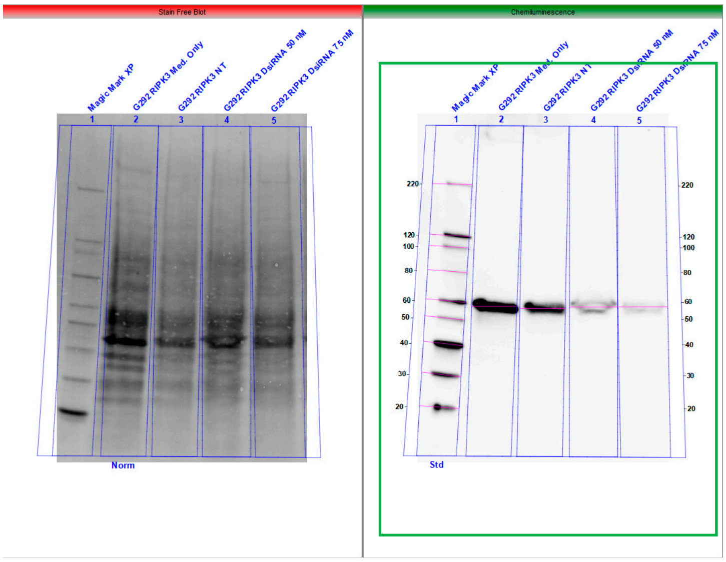

Figure 5A: 143B cells stably expressing RIPK3 demonstrate elevate levels of phosphorylated RIPK3 (p-RIPK3; 87 kDa; Top image) following cisplatin treatment compared to EV controls. Total RIPK3 is shown below on a new gel using the same protein lystaes. Bands within the green box used in manuscript figure.

p-RIPK3:

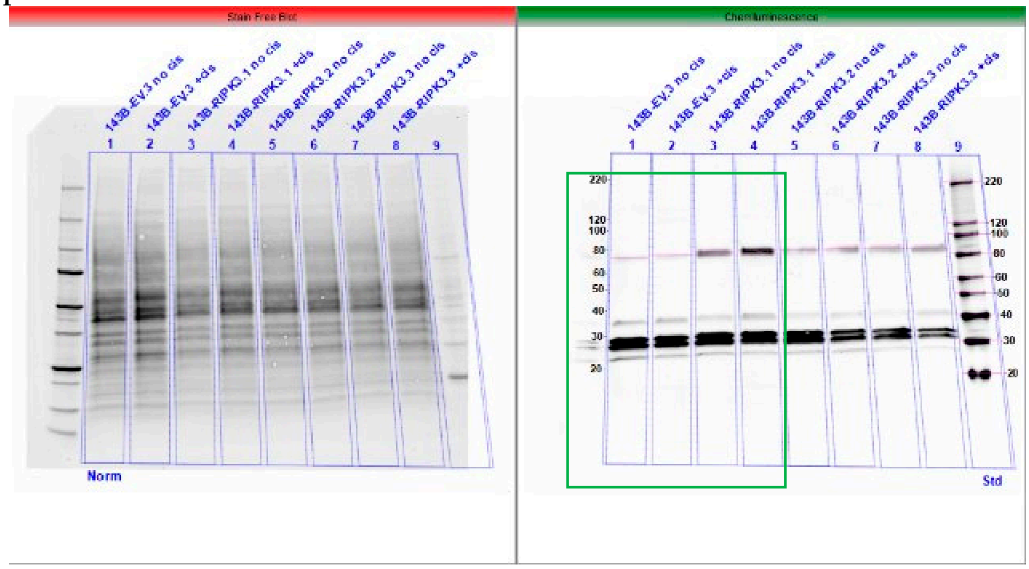

Total RIPK3:

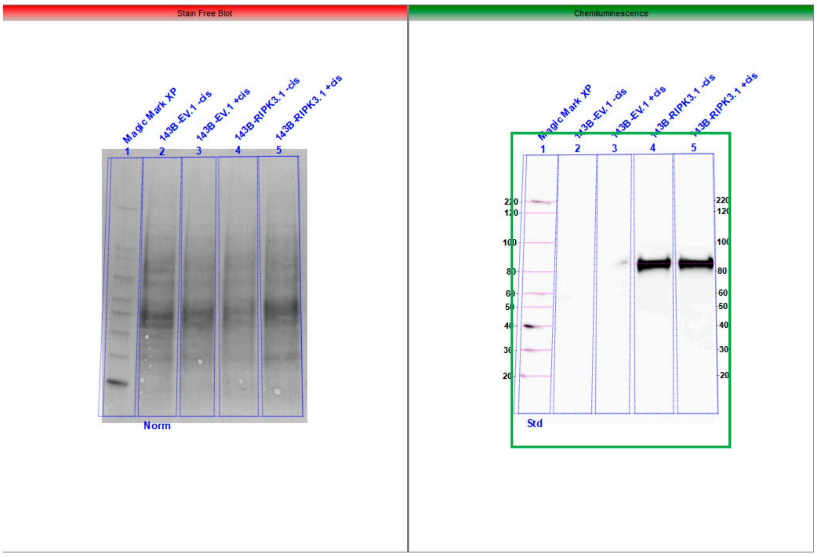

Figure 5C: RIPK3 expressing G292 cells also show a significant increase in p-RIPK3 (60 kDa; Top image) levels compared to EV and untreated controls. Total RIPK3 is shown on the second image using the same protein lysates. Bands within the green box used in manuscript figure.

p-RIPK3:

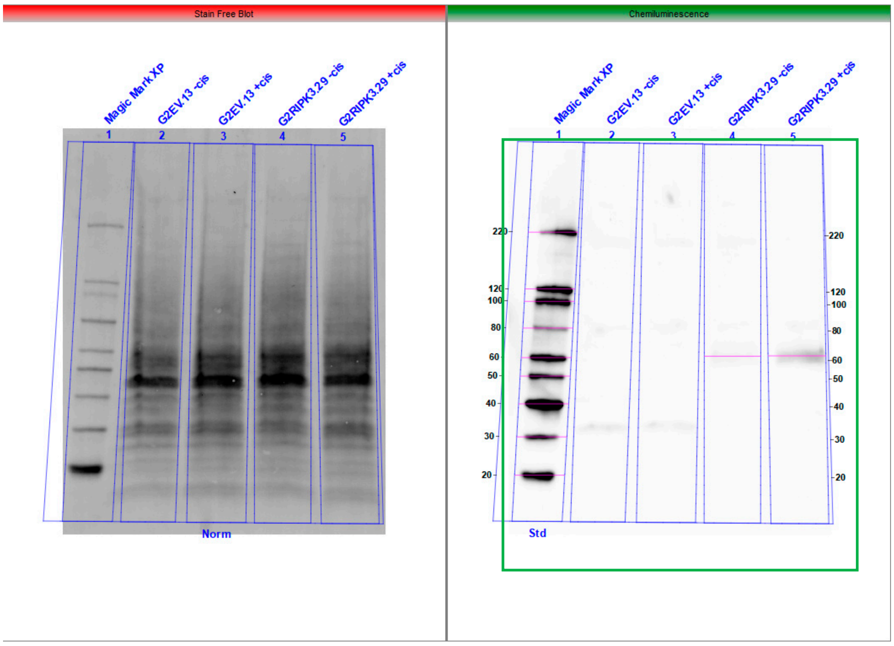

Total RIPK3:

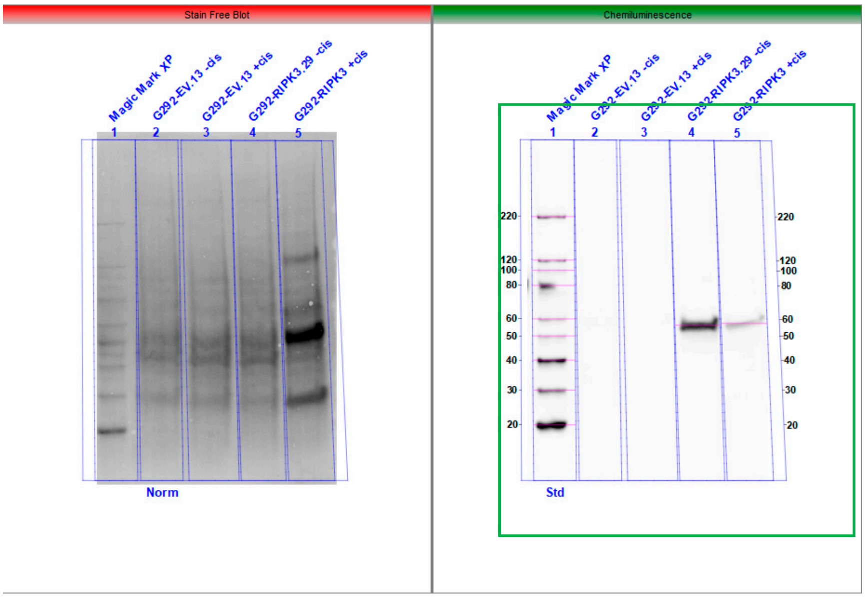

Figure 5F: 143B cells stably expressing RIPK3 show significantly elevated levels of phosphorylated MLKL (p-MLKL; 54 kDa; Top image) following both 24- and 48 hrs cisplatin treatment compared to EV and untreated controls. Total MLKL (54 kDa) is shown on the same blot in the bottom image. Bands within the green box used in manuscript figure.

p-MLKL:

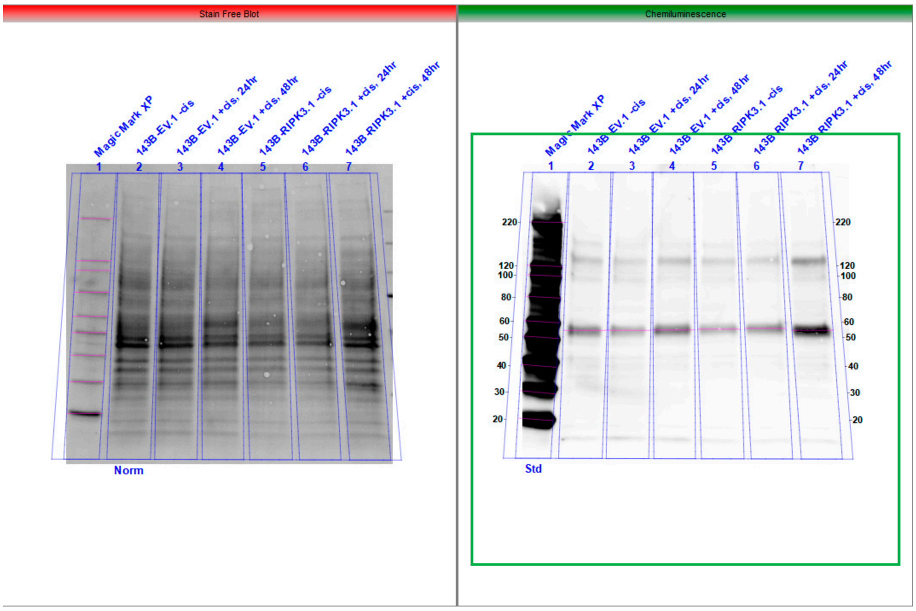

Total MLKL:

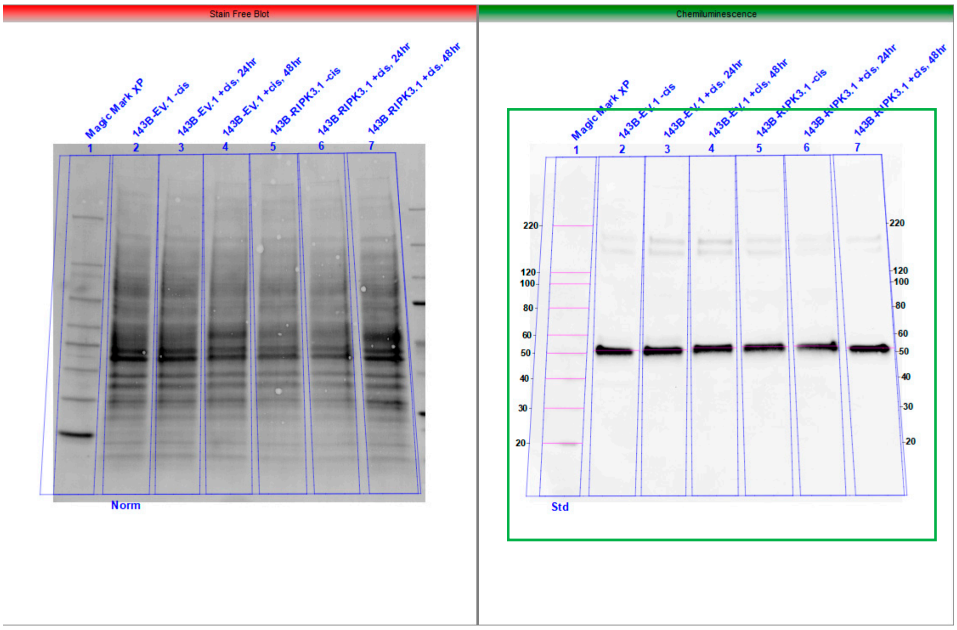

	**Mean Percent Methylation-MLKL**
Sample	NGS
hMSC	53.8
hFOB	50.5
HOS	55.6
G292	56.4
MNNGHOS	56.3

MG63	56.7
143B	56.0
Non-cancerous bone 1	0.6
Non-cancerous bone 2	54.2
OS 1	53.4
OS 2	54.0
OS 3	54.2
OS 4	54.7
OS 5	47.3
OS 6	-
OS 7	55.2
OS 8	50.2
OS 9	50.8
OS 10	55.7
OS 11	48.9
NGS Methylation Control 0%	1.1
NGS Methylation Control 10%	9.0
NGS Methylation Control 25%	20.9
NGS Methylation Control 50%	39.4
NGS Methylation Control 75%	60.0
NGS Methylation Control 90%	75.2
NGS Methylation Control 100%	94.2
No template control	0.0

Table S1: Percent mean methylation of MLKL in cell lines, healthy human controls and OS tumor samples resected after induction chemotherapy. Percent methylation was assessed by next generation sequencing (NGS). Samples analyzed included several cell types (human mesenchymal stem cells (hMSC), human fetal osteoblasts (hFOB)), OS cell lines (HOS, G292, MNNGHOS, MG63, 143B), two non-cancerous bone samples, OS tumor samples (n=11), and Pyrosequencing or NGS controls.
